# Levels of CD105^+^ cells increase and cell proliferation decreases during S-phase arrest of amniotic fluid cells in long-term culture

**DOI:** 10.3892/etm.2014.1959

**Published:** 2014-09-12

**Authors:** DING WANG, RUI CHEN, XUAN ZHONG, YONG FAN, WEIQIANG LAI, XIAOFANG SUN

**Affiliations:** 1 Key Laboratory for Major Obstetric Diseases of Guangdong Province, The Third Affiliated Hospital of Guangzhou Medical University, Guangzhou, Guangdong 510150, P.R. China; 2 Key Laboratory of Reproduction and Genetics, Guangdong Higher Education Institutes, The Third Affiliated Hospital of Guangzhou Medical University, Guangzhou, Guangdong 510150, P.R. China; 3 Experimental Department, Institute of Gynecology and Obstetrics, The Third Affiliated Hospital of Guangzhou Medical University, Guangzhou, Guangdong 510150, P.R. China

**Keywords:** amniotic fluid cells, CD105, cell proliferation, long-term culture, S-phase arrest

## Abstract

The present study aimed to improve the characterization of amniotic fluid cells (AFCs) in order to optimize their use in chromosomal prenatal diagnosis and as seed or stem cells for tissue engineering. The AFCs used in the current study were obtained from three females in their second trimester of pregnancy. The cells were cultured independently and characterized by cell morphology, cell markers, cell cycle distribution and chromosome Giemsa banding in an early- and late-passage. The AFCs remained homogeneous in culture and expressed mesenchymal markers, but not endothelial markers along the culture process. In addition, compared with the early-passage cells, the late-passage cells exhibit an increase in CD105 expression, a decrease in cell division and a delay in the cell cycle, and a number of cells underwent cell cycle arrest. However, the cells retained a normal karyotype. Therefore, the current study characterized AFCs in a clinical culture and confirmed that AFCs are mesenchymal precursors. The results obtained may be useful for the application of AFCs in prenatal diagnosis.

## Introduction

Amniotic fluid contains a number of living cells that have undergone defluxion from the fetus and fetal membranes (1). As the amniotic fluid cells (AFCs) share the same genomic background as the fetus, they represent an important prenatal diagnosis target (2–8). Amniotic fluid has been used for diagnosing genetic disease (8), cytogenetic analysis (5,7) and comparative genomic hybridization (CGH) assays (6). As the process of amniocentesis causes trauma, a degree of maternal blood interference is unavoidable. Since blood cells are unable to proliferate or adhere in culture in the absence of stimulus factors, it is necessary to optimize the culture to specifically amplify fetal cells in order to minimize maternal interference, collect metaphase cells and acquire sufficient genomic DNA.

Previous studies have focused on stem cells derived from amniotic fluid. Initially, AFCs were identified as glial cells and monocyte-derived macrophages (9). Furthermore, it was presumed that mesenchymal stem cells were derived from the AFCs (10) on the basis of their cell morphology, specific surface markers and their capacity for neural, adipogenic and osteogenic differentiation. A study by De Coppi *et al* maintained pluripotent-specific cells in pluripotent stem cell culture medium. These cells were positive for CD markers, expressed pluripotent stem cell markers and were able to differentiate into three germ lines (11). Additional studies have demonstrated that AFCs are easily reprogrammed into induced pluripotent stem cells (iPSCs) (12–14). As a result of their ability to self-renew and differentiate into functional somatic cells, amniotic fluid-derived stem cells have been used as seed cells for tissue engineering (11,15,16) and iPSCs have been used to model genetic disease (13,17).

Due to the wide application of AFCs in prenatal diagnosis and their potential use as stem cells, the amplification and identification of the characteristics of AFC cultures is important for clinical laboratory diagnosis and biological study. In the present study, surface markers, chromosome Giemsa (G)-banding, cell cycle distribution and the proliferation of AFCs in an early- and late-passage were assessed.

## Materials and methods

### Cell culture

Three independent amniotic fluid samples from two females at 20 weeks and one female at 21 weeks into the gestation period, who took part in a clinical cytogenetic diagnosis, were included in the study. The patients provided consent and the study was approved by the Ethics Committee of The Third Affiliated Hospital of Guangzhou Medical University (Guangzhou, China). A total of 16 ml amniotic fluid was extracted from each patient by amniocentesis for cytogenetic diagnosis. Each sample was separated into two tubes of 8 ml each. Following centrifugation at 300 × g for 5 min, the cell precipitate in each tube was collected for culture in a 25 cm^2^ flask [passage (P)0; Gibco AmnioMAX^™^-C100; Invitrogen Life Technologies, Carlsbad, CA, USA]. The cells were passaged for nine days following inoculation in 0.05% trypsin-EDTA (Invitrogen Life Technologies). Subsequently, one tube was seeded into an additional 25 cm^2^ flask for G-banding analysis, while the other tube was seeded into a 75 cm^2^ flask for further experiments. The subcultured cells were passaged four times every 5–6 days, when 80–90% of cells were confluent. Cells from P1 and P6 were regarded as early- and late-passages, respectively, and their analyses were compared.

### G-banding

Cultured AFCs were incubated in 25 mg/ml colchicine for 4 h and harvested using 0.05% trypsin-EDTA. The cells were made hypotonic using hypotonic medium [0.4% sodium citrate combined with 0.4% potassium chloride (1:1)], fixed in a fixation medium [acetic acid combined with methanol (1:3)], dropped on precooled glass and cultured at 60°C overnight. G-banded karyotyping was performed by digesting the cells with trypsin, followed by staining with a Giemsa stain. Images were captured and analyzed using an Ikaros system (Carl Zeiss AG, Oberkochen, Germany).

### Proliferation curve rendering

For each individual culture, 1×10^5^ cells were seeded into nine independent 9.6 cm^2^ wells to analyze their proliferative potential. After 2 h allowing for adherence, cells from three wells were trypsinized and counted at 24, 48 and 72 h. As there were no statistically significant difference between each individual. The mean values and standard deviations of each time were used to construct a proliferation curve. This experimental protocol was performed for P1 and P6 cells.

### Flow cytometry analysis

Adherent AFCs were separated by trypsin treatment and fixed in 75% ethanol overnight at 4°C. The cells were filtered through a 40 μm mesh and resuspended in fluorescence-activated cell sorting (FACS) buffer [phosphate-buffered saline (PBS) containing 2% fetal bovine serum (FBS) and 0.1% sodium azide]. Directly-conjugated isotype control antibodies, IgG-fluorescein isothiocyanate (FITC) and IgG-phycoerythrin (PE; BD Pharmingen, San Diego, CA, USA), were used as controls to identify the background cells. A total of ~5×10^5^ cells were incubated at 4°C for 40 min with each of the following FITC- or PE-conjugated antibodies (BD Pharmingen): CD133-PE, CD117-PE, CD34-PE, CD105-FITC, CD106-FITC, CD29-PE, CD44-FITC, CD147-FITC and CD90-PE. The cells were subsequently washed in FACS buffer. The antibody-labeled cells were analyzed using a BD FACSCalibur instrument (BD Biosciences, Franklin Lakes, NJ, USA), and data were analyzed using FlowJo version 7.2.5 software (TreeStar, Inc., Ashland, OR, USA). The cell count experiments for the flow cytometry assays were performed in triplicate.

### Cell cycle distribution

AFCs were harvested and fixed using the same method as described previously for flow cytometry analysis. Cellular DNA was stained with propidium iodide (PI) at 4°C for 30 min in a staining solution composed of PBS with 50 μg/ml PI, 100 μg/ml RNase A and 0.2% Triton X-100. The cells were counted using a BD FACSCalibur instrument. ModFit software (BD Biosciences) was used to conduct analysis.

### Statistical analysis

For P1 and P6, nine independent cultures of each AFC were performed. The nine cultures were divided into three groups, three cultures were harvested at 24, three at 48 and three at 72 h. Differences between the individual cultures were analyzed using a t-test. In addition, the CD^+^ percentage and percentage of AFCs in each cell cycle stage for the early- and late-passage cultures were compared using the t-test. All statistical analyses were performed by SPSS statistical software (version 19.0, IBM SPSS Inc., Chicago, IL, USA). P<0.05 was considered to indicate a statistically significant difference.

## Results

### AFCs exhibit different morphologies in primary culture

**Figure 1:**
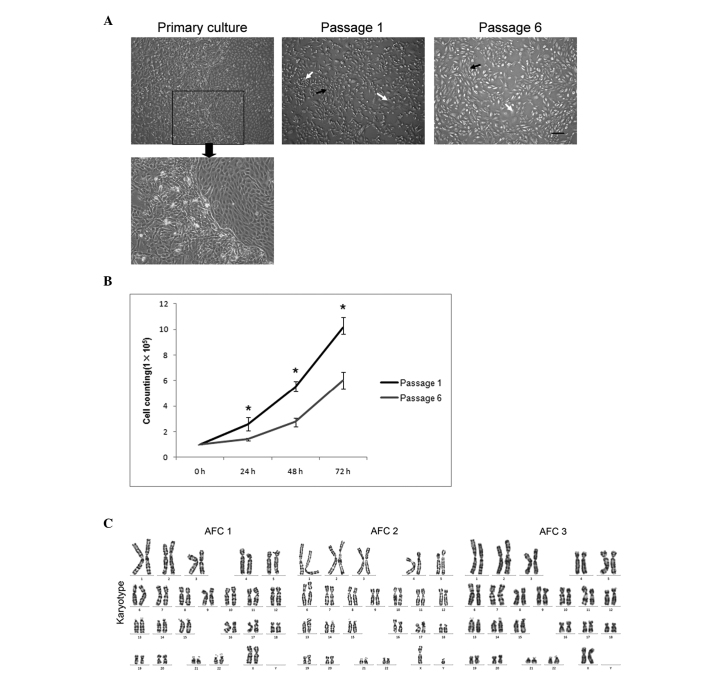
Cell morphology of AFCs in long-term culture reveal a normal karyotype. (A) Endothelial and fibroblast colonies are apparent in the primary culture, with a number of endothelial (white arrow) and fibroblast (black arrow) cells present in the subculture. For the first line figure, bar=50 μm, for the second line figure, bar=25 μm. The first line figure magnification, ×50; the second line figure magnification, ×100. (B) Proliferation curves for passages 1 and 6. ^*^P<0.05 vs. passage 6. (C) AFCs retained a normal karyotype in passage 6. AFC, amniotic fluid cells.

Following seven days of primary culture, a number of cells adhered to form cell colonies, whereas other cells remained suspended and were removed when the medium was changed. The cell colonies were divided into two types according to their morphology: Endothelial and fibroblast-like. Endothelial colonies were characteristically polygonal, large cells with a tight cell connection and a legible colony edge. By contrast, the fibroblast-like colonies comprised slender, spindle-shaped cells that did not possess a tight connection and typically exhibited a number of cells away from the colony edge (Fig. 1). After nine days of primary culture, as the fibroblast cells grew more rapidly than the endothelial cells, certain fibroblast-like colonies became confluent with one other or with the endothelial colonies. At this stage, the primary culture cells were trypsinized and termed as P1.

### Early-passage AFCs exhibit a mesenchymal cell marker and a normal karyotype

**Figure 2:**
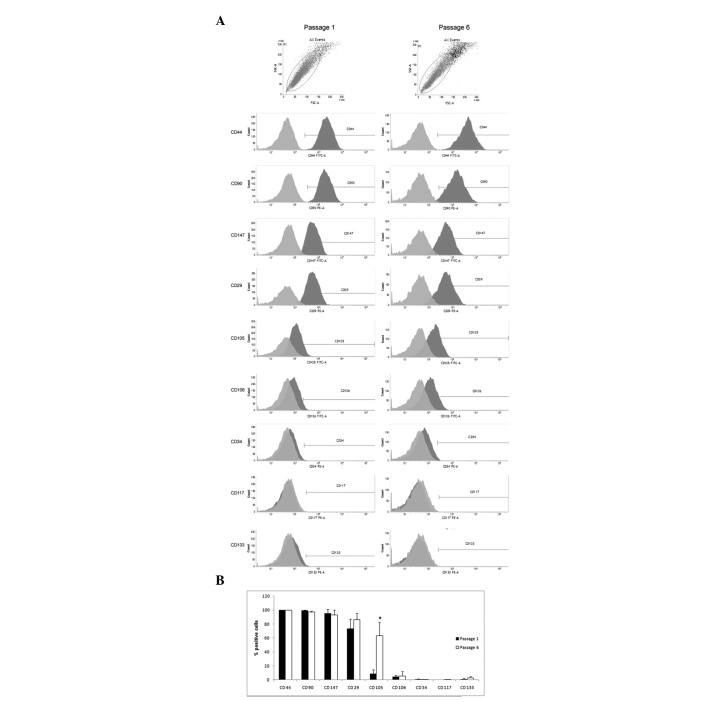
Flow cytometric analysis of the AFC subculture. (A) The positive cell percentage of each marker was calculated by flow cytometric analysis. The flow cytometric analysis of AFC1 is shown. (B) Comparison of the positive cell percentages between passages 1 and 6 for each marker. ^*^P<0.05, vs. passage 1. AFC, amniotic fluid cell.

**Figure 3:**
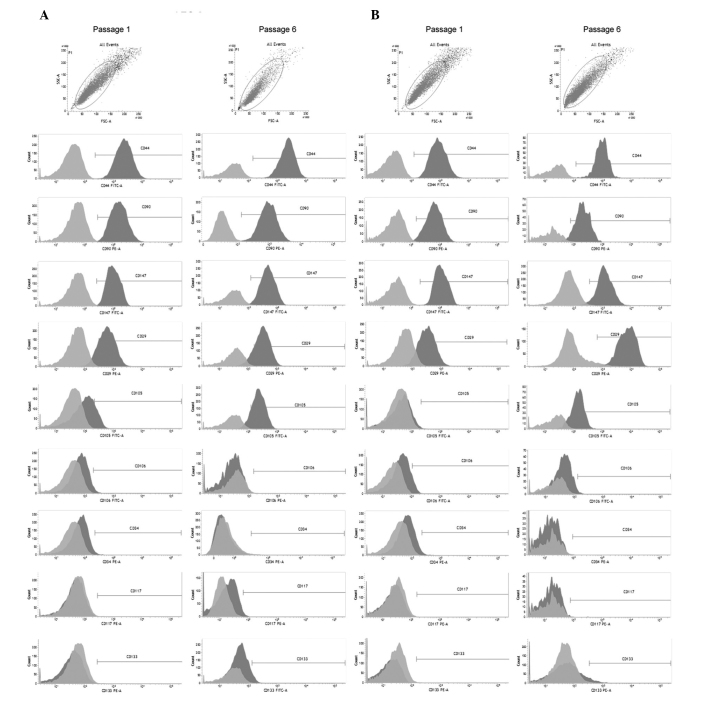
Flow cytometry analysis of the (A) AFC2 and (B) AFC3.

**Figure 4:**
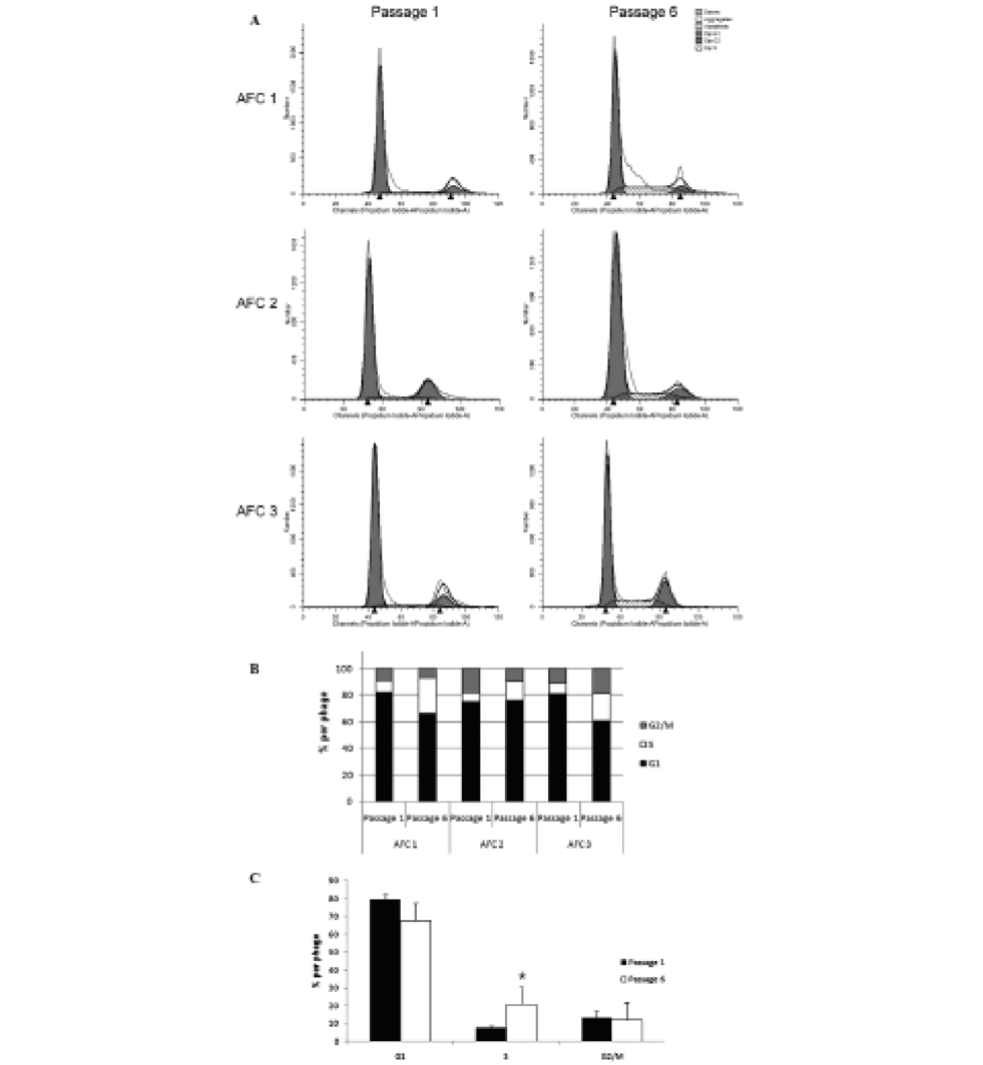
Cell cycle distribution of the AFC subculture. (A) DNA content analysis using flow cytometry. (B) Cell cycle stage ratios for each measurement. (C) Comparison of the cell cycle stage ratios between passages 1 and 6. AFC, amniotic fluid cell.

Following the primary AFC subculture, the adherent cells remained distinguishable as fibroblast or endothelial cells, and certain small endothelial colonies were apparent. A number of the P1 cells were harvested for karyotype G-banding, cell surface marker or cell cycle distribution by flow cytometry analyses. The remaining cells were cultured for cell proliferation analysis and long-term culture. Although the percentage of CD^+^ cells varied among the three independent cultures, the P1 cells were almost all (>85%) positive for the mesenchymal cell markers, CD44, CD90 and CD147, partially positive for CD29, CD105 and CD106, but negative (<1.5%) for CD117, CD34 and CD133 (Figs. 2 and 3). G-banding analysis revealed that the three AFC cultures exhibited normal karyotypes (Fig. 1) and were at the following cell-cycle stages: G1 (79.27%), S (7.63%) and G2/M (13.1%; Fig. 4). The proliferative ability was obtained by quantifying the number of cells in culture, and statistically significant differences were not observed among the AFC cultures following t-test analysis. The time taken for the cells to double in quantity was 24 h (Fig. 1).

### Late-passage AFCs retain a normal karyotype, but cell surface markers change, cell cycle is blocked in S-phase and proliferation ability is reduced

The late-passage of AFCs refers to cells that have undergone six passages, and during the nearly 30 day culture there was 30 times doubling. G-banding analysis revealed that the three AFC groups maintained a normal karyotype (Fig. 1). With regard to morphology, the tight endothelial colonies did not exist, and the cells exhibited a homogeneous morphology. The late-passage AFCs were similar to the early-passage AFCs in that they expressed almost all the same mesenchymal cell markers, including CD44, CD90 and CD147, were partially positive for CD29, CD105 and CD106, but negative for CD117, CD34 and CD133. However, the percentage of CD105^+^ cells in the late-passage AFC cultures was significantly higher compared with the early-passage cells (P<0.05; Fig. 2 and 3). The AFCs exhibited the following cell cycle stage distributions: G1 (67.59%), S (20.49%) and G2/M (11.91%). On average, the percentages of late-passage AFCs in the G1 and G2/M phases were not significantly different when compared with the early-passage AFCs; however, the percentage of cells in the S-phase was significantly higher in the late-passage cells compared with the early-passage cells (P<0.05; Fig. 4). The proliferative ability of the AFCs exhibited statistically significant differences between the passages when the doubling time was 24–48 h (Fig. 1).

## Discussion

AFC cultures are not only required for traditional prenatal diagnosis, but are also widely used for purifying fetal samples in order to avoid interference from maternal material when genetically diagnosing diseases or performing CGH assays (2,3,6–8). Furthermore, a number of studies on stem cells and tissue engineering have indicated that AFCs are suitable for acquiring human genetic material for disease diagnosis (12,13) and for use as seed cells in tissue engineering (11,15,16). In the present study, three AFC samples from pregnant females in their second trimester were included for a duration that was suitable for prenatal diagnosis based on amniotic fluid (18–20).

Previously, primary AFCs were regarded as colonial-morphology growth cells, while fetal urine was considered a potential source of AFCs (18,19). The morphology of the colonies was separated into two or three divisions. The two divisions were F-type (identical to cultured human dermal fibroblasts) and AF-type (amniotic fluid type), characterized by the presence of type I collagen fibers (1), while the three division were fibroblast-like (F), epithelioid (E) and clonable AFCs (20). In the current study, the colonies in the primary culture were divided into endothelial or fibroblast-like colonies; all of which originated from a fetal source and were able to proliferate.

As the amount of primary AFC culture acquired was not sufficient to perform all the subsequent analyses, the AFCs were subcultured. In the subculture, few suspended cells were discarded and the incubation time from primary culture to P1 was 48 h. The P1 cell type was considered to be similar to the primary culture. A clinical cytogenetic diagnosis was performed on the P1 cells and the three independent cultures were demonstrated to exhibited a normal karyotype. Flow cytometry analysis results indicated that almost all the cells were positive for CD44, CD90 and CD147 and almost all were negative for CD34, CD117 and CD133. A number of the AFC population was positive for CD29, CD105 and CD106, with a variety of frequencies for each. The clinically cultured AFCs were positive for mesenchymal cell markers and negative for endothelial, hematopoietic and stem cells markers. This observation may be due to the culture medium and state, which is consistent with the results of a previous study on cell phenotypes (21,22). Several studies have derived hematopoietic stem cells from amniotic fluid and performed screening using flow cytometry prior to establishing the primary culture (11). These cells were discarded in the primary culture in the current study. The AFCs were able to proliferate well and doubled in abundance within 24 h; on average, 13.1% of the culture was in the mitotic phase. The main purpose for clinically culturing AFCs has been for cytogenetic analysis (5,20). In the present study, the clinical culture conditions were suitable for this purpose as there were a sufficient number of cells in the G2/M phase.

The present subculture was based on cell growth, where following six passages and almost 30 divisions, the AFCs retained a normal karyotype. The identification experiment was performed by long-term culture comparison. With regard to the cell surface markers, only the percentage of CD105^+^ cells significantly increased in the late-passage when compared with the early-passage AFC cultures; the other markers were not significantly changed. Considering that CD105 is a mesenchymal marker, the long-term culture conditions are permissive for mesenchymal cell growth or for mesenchymal progenitor cells to differentiate into CD105^+^ cells. Furthermore, the clinical culture system was similar to a previously published amniotic fluid stem cell study system (23), where amniotic fluid stem cells were derived to perform adipogenic, osteogenic, neurogenic and myogenic differentiation. These cells also expressed mesenchymal and neural markers, but not hematopoietic markers. In the present study, the late-passage cells proliferated at a slower rate compared with the early-passage cells, evident from the doubling time, which was >24 h but <48 h, but the doubling time was 24 h for the early-passage cells. There were a sufficient number of mitotically dividing cells to conduct karyotype analysis, consistent with the 11.91% of cells in a mitotic stage. Cell cycle distribution analysis indicated that the percentage of cells in the S-phase significantly increased during the late-passage, whereas the percentage at other stages were not significantly different. It was hypothesized that the cells were proliferating slowly due to S-phase arrest in the late-passage cells.

In conclusion, the present study characterized the exchange of surface markers and cell cycle distribution in an AFC culture. It demonstrated hat the number of CD105^+^ AFCs increased following long-term culture and the AFC proliferation decreased due to cell cycle arrest in the S-phase. As the AFC culture was widely used in tissue engineering and clinical prenatal diagnosis, our study would be useful in the specific mesenchymal cell acquisition and prenatal diagnosis sample keeping.

## References

[1] Priest RE, Marimuthu KM, Priest JH (1978). Origin of cells in human amniotic fluid cultures: ultrastructural features. Lab Invest.

[2] Macek M, Hurych J, Hyánek J (1971). Cultivation of amniotic fluid cells and prenatal diagnosis. Cesk Gynekol.

[3] Mulcahy MT, Jenkyn J (1973). Prenatal diagnosis. Results of cytogenetic analysis of amniotic fluid cell cultures. Med J Aust.

[4] Macek M, Suk V, Bresták M, Rezácová D, Houstĕk J, Kotásek A (1974). Cultivation of cells from amniotic fluid for prenatal diagnosis. Cesk Pediatr.

[5] Zolotukhina TV (1980). Amniotic fluid cell culture for the prenatal diagnosis of the fetal karyotype. Tsitol Genet.

[6] Lapierre JM, Cacheux V, Collot N (1998). Comparison of comparative genomic hybridization with conventional karyotype and classical fluorescence in situ hybridization for prenatal and postnatal diagnosis of unbalanced chromosome abnormalities. Ann Genet.

[7] Guven MA, Ceylaner G, Coskun A (2006). Volume of sampled amniotic fluid and prenatal cytogenetic diagnosis. Int J Gynaecol Obstet.

[8] Rebello MT, Hackett G, Smith J (1991). Extraction of DNA from amniotic fluid cells for the early prenatal diagnosis of genetic disease. Prenat Diagn.

[9] Polgár K, Adány R, Abel G, Kappelmayer J, Muszbek L, Papp Z (1989). Characterization of rapidly adhering amniotic fluid cells by combined immunofluorescence and phagocytosis assays. Am J Hum Genet.

[10] In ‘t Anker PS, Scherjon SA, Kleijburg-van der Keur C (2003). Amniotic fluid as a novel source of mesenchymal stem cells for therapeutic transplantation. Blood.

[11] De Coppi P, Bartsch G, Siddiqui MM (2007). Isolation of amniotic stem cell lines with potential for therapy. Nat Biotechnol.

[12] Li C, Zhou J, Shi G (2009). Pluripotency can be rapidly and efficiently induced in human amniotic fluid-derived cells. Hum Mol Genet.

[13] Fan Y, Luo Y, Chen X, Li Q, Sun X (2012). Generation of human β-thalassemia induced pluripotent stem cells from amniotic fluid cells using a single excisable lentiviral stem cell cassette. J Reprod Dev.

[14] Li Q, Fan Y, Sun X, Yu Y (2013). Generation of induced pluripotent stem cells from human amniotic fluid cells by reprogramming with two factors in feeder-free conditions. J Reprod Dev.

[15] Mirabella T, Cilli M, Carlone S, Cancedda R, Gentili C (2011). Amniotic liquid derived stem cells as reservoir of secreted angiogenic factors capable of stimulating neo-arteriogenesis in an ischemic model. Biomaterials.

[16] Mirabella T, Poggi A, Scaranari M (2011). Recruitment of host’s progenitor cells to sites of human amniotic fluid stem cells implantation. Biomaterials.

[17] Lu HE, Yang YC, Chen SM (2013). Modeling neurogenesis impairment in Down syndrome with induced pluripotent stem cells from trisomy 21 amniotic fluid cells. Exp Cell Res.

[18] Hoehn H, Bryant EM, Karp LE, Martin GM (1974). Cultivated cells from diagnostic amniocentesis in second trimester pregnancies. I Clonal morphology and growth potential. Pediatr Res.

[19] Hoehn H, Bryant EM, Fantel AG, Martin GM (1975). Cultivated cells from diagnostic amniocentesis in second trimester pregnancies. III The fetal urine as a potential source of clonable cells. Humangenetik.

[20] Hoehn H, Bryant EM, Karp LE, Martin GM (1975). Cultivated cells from diagnostic amniocentesis in second trimester pregnancies. II Cytogenetic parameters as functions of clonal type and preparative technique. Clin Genet.

[21] Davydova DA, Vorotelyak EA, Smirnova YA (2009). Cell phenotypes in human amniotic fluid. Acta Naturae.

[22] Cananzi M, De Coppi P (2012). CD117(+) amniotic fluid stem cells: state of the art and future perspectives. Organogenesis.

[23] Bossolasco P, Montemurro T, Cova L (2006). Molecular and phenotypic characterization of human amniotic fluid cells and their differentiation potential. Cell Res.

